# Midwives and public health nurses' knowledge and clinical practice in securing sufficient iodine status in relation to pregnancy: A cross‐sectional study

**DOI:** 10.1002/nop2.1675

**Published:** 2023-02-25

**Authors:** Maren Johnsen, Tonje Braaten, Guri Skeie, Hilde Laholt, Solrunn Hansen

**Affiliations:** ^1^ Department of Health Care and Sciences. Faculty of Health Sciences UiT ‐ The Arctic University of Norway Tromsø Norway; ^2^ Department of Community Medicine, Faculty of Health Sciences UiT ‐ The Arctic University of Norway Tromsø Norway

**Keywords:** dietary, infant, iodine, lactation, maternal health, midwifery, nutrition, pregnancy, public health nurses

## Abstract

**Aim:**

To investigate midwives' (MWs) and public health nurses' (PHNs) clinical practice and knowledge related to nutrition, with a particular focus on iodine in northern parts of Norway. Maternal iodine status prior to and during pregnancy, and the lactating period, is crucial for brain development and growth of the foetus and infant, from conception up until the first two years of life. In Norway, studies have documented mild to moderate iodine deficiency in this group.

**Design/Methods:**

MWs (*n* = 128) and PHNs (*n* = 154) responded to a survey regarding nutrition and iodine. Descriptive data and non‐parametric tests were used to analyse data.

**Results:**

Around half of the participants provided dietary guidance to a great extent. Practice of iodine‐specific recommendations was lower, particularly regarding lactating women. Compared to other nutrients, iodine was not a priority.

**Conclusion:**

The study indicates a lack of knowledge and poor clinical practice about iodine among MWs and PHNs.

## 1 INTRODUCTION

Maternal iodine status in pre‐pregnancy, pregnancy and the lactating period is crucial to the brain development and growth of the foetus and infant (WHO, 2007; Zimmermann, 2009). The latest estimate is that 1.88 billion people globally have insufficient dietary iodine intake (Andersson et al., 2012). In Norway, recent, studies have documented mild to moderate iodine deficiency in young fertile women, and pregnant and lactating women (Aakre et al., 2020; Berg et al., 2017; Groufh‐Jacobsen et al., 2020; Henjum et al., 2018; Henjum et al., 2019; Næss et al., 2021). Concurrently, a low level of knowledge among the fertile female population of the importance of iodine, dietary sources and daily recommended intake of iodine has been documented (Garnweidner‐Holme et al., 2017; Groufh‐Jacobsen et al., 2020; Henjum et al., 2018). The Norwegian National Council for Nutrition concluded that iodine deficiency is present in these vulnerable groups and that due to their competence and practice, healthcare professionals have a special responsibility to ensure an adequate iodine status (Norwegian National Council for Nutrition, 2016).

In the Norwegian healthcare system, midwives (MWs) and public health nurses (PHNs) play an important role in the lives of women and children (Norwegian Directorate of Health, 2014, 2017, 2018). Midwives care for women during pregnancy, birth, and the postnatal period. In consultations, information about health and lifestyle is offered during the first trimester, including dietary and nutritional recommendations (Norwegian Directorate of Health, 2018). Public health nurses specialise in health promotion and disease prevention for children (0–20 years) and their families. They care for child and adolescent health, as well as maternal health, mostly postnatal (Norwegian Directorate of Health, 2014, 2017, 2018). Child Health Clinics target 0–5 year‐old children through statutory consultations, including health dialogue about diet and nutrition. School health services (5–20 years) and adolescent health clinics support the youth and the adolescent population and offer lifestyle and dietary recommendations (Norwegian Directorate of Health, 2017). All municipalities are responsible for managing a PHN service (Helse‐ og omsorgstjenesteloven, 2011). Pending a PHN, a registered nurse is temporarily employed as a PHN, hereafter called ‘constituted PHN’. Both MWs' and PHNs' ideologies and guidelines are based on principles of user involvement and empowerment (ICM, 2008; ICN, 2011; Norwegian Directorate of Health, 2014, 2017, 2018). Dietary recommendations are based on the Norwegian dietary guidelines (Norwegian Directorate of Health, 2011).

In pregnancy guidelines, essential nutrients such as folate, iron, calcium, vitamin B12 and vitamin D have been highlighted for many years, but iodine was only included in 2018 in a revised version of the guidelines after a report from the Norwegian National Council for Nutrition from 2016 (Norwegian Directorate of Health, 2018; Norwegian National Council for Nutrition, 2016). The postnatal care guidelines do not include dietary or nutritional recommendations (Norwegian Directorate of Health, 2014). Guidelines for infants emphasise iron and vitamin D, while monitoring iodine is included from the age of 6–11 months (Norwegian Directorate of Health, 2016;2019).

An unbalanced diet or reduced iodine levels in food entail a risk of iodine deficiency (Zimmermann, 2009). Important Norwegian food sources are fish, seafood, eggs, cow's milk, hereafter called milk, and dairy products, with the latter two contributing around 60% of iodine intake. No iodine fortification programme exists in Norway (Norwegian National Council for Nutrition, 2016). Maternal iodine requirements are increased during pregnancy, with the embryonic period as the most critical. Iodine deficiency in the preconception period thus challenges maternal and foetal health (Zimmermann, 2009). Also, exclusively breastfed infants are entirely dependent on iodine supplied via breast milk, to cover their high rates of thyroid hormone production (Andersson et al., 2007). For the child, maternal iodine deficiency may have serious to less serious health effects on brain development and cognition: intellectual disability, decreased IQ, and in the worst case induces cretinism (Zimmermann, 2011). The brain needs iodine for its development up until the first 2 years of life (WHO, 2007).

Iodine deficiency is simple to prevent, with a low‐cost intervention (Zimmermann, 2009). Healthcare professionals such as MWs and PHNs are in a unique position to contribute knowledge and ensure adequate iodine intake in relation to preconception, maternal and postnatal needs (Arrish et al., 2016; Bouga et al., 2018; Norwegian National Council for Nutrition, 2016; Othman et al., 2020). Studies highlight the need to improve healthcare providers' clinical practice and education concerning nutrition and diet in general and in particular related to iodine (Arrish et al., 2014; Arrish et al., 2016; Bryant et al., 2019; Charlton et al., 2012; Guess et al., 2017; Lee et al., 2018; Lucas et al., 2014; Malta et al., 2016; Soltani et al., 2017). To the best of our knowledge, no previous research studies of Norwegian healthcare professionals' clinical practice concerning iodine exist. In light of iodine deficiency and less satisfactory iodine knowledge in reproductive target groups, both in Norway and other countries (Aakre et al., 2020; Berg et al., 2017; Brantsæter et al., 2013; Candido et al., 2019; Charlton et al., 2012; Combet et al., 2015; Groufh‐Jacobsen et al., 2020; Henjum et al., 2017; Henjum et al., 2018; Jiang et al., 2019; Nyström et al., 2016; Patriota et al., 2022; Zimmermann et al., 2015), we hypothesise that there is limited knowledge about and focus on iodine in Norwegian midwifery and public health nurses' clinical practice.

This study targets midwives and public health nurses in northern parts of Norway. The aim is to investigate clinical practice and knowledge regarding nutrition with a special focus on iodine.

## 2 MATERIALS AND METHODS

This cross‐sectional study is part of the MISA study at UiT The Arctic University of Norway focusing on diet, lifestyle factors and persistent toxic elements related to reproductive health in women living in selected areas of northern Norway. Midwives and public health nurses operating in public services in northern Norway were invited to respond to a survey of clinical practice concerning these topics. The present sub‐study investigates practice and knowledge about diet, with a focus on iodine.

Prior to the survey, all the special clinics, hospital maternity wards and midwifery‐led units (*n* = 15) (hereafter named maternity wards) and child health clinics (*n* = 84) in northern Norway were contacted. With the exception of one child health clinic with a partly reported number of personnel, all institutions reported the total number of target personnel serving young, fertile, pregnant and lactating women. Invitation letters with unique participant numbers and pin codes to a web‐based questionnaire were delivered to the service units for internal distribution. For promotion, posters and repeated emails to target personnel were utilised. The study period was nine weeks, from December 2017 until January 2018.

### 2.1 Population and sample

**FIGURE 1:**
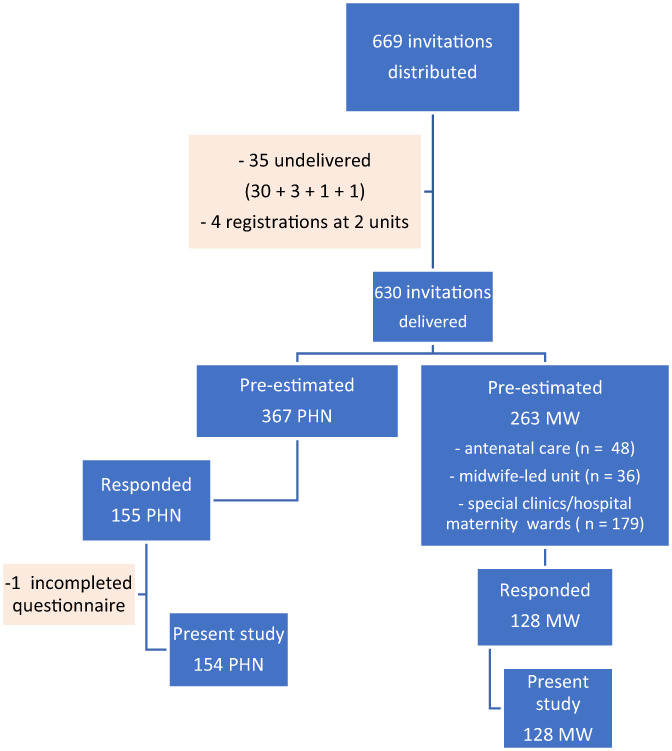
Overview of the study group of midwives and public health nurses operating in public clinical practice servicing fertile young women, and pregnant and lactating women in northern Norway, for the year from December 2017 to January 2018. Midwife, MW; public health nurses; PHN.

A total of 669 invitation letters were distributed, of which 35 were reported to be internally undelivered and four participants were registered at two units. Among the 630 eligible for participation, 128 (49%) MWs and 155 (42%) PHNs answered the questionnaire. One of the PHNs was excluded from the survey due to an incomplete questionnaire. Thus, 282 participants were included in the present study (Figure 1). Of these, six had a position as both a MW and PHN. For convenience, and based on education, healthcare institution and percentage employment, these participants were classified as either MW (*n* = 2) or PHN (*n* = 4).

### 2.2 Questionnaire

From the entire questionnaire comprising 56 questions, the present study was restricted to 32 questions explaining background variables, and dietary and specifically iodine clinical practice and knowledge.

Demographic characteristics were described: age, years of professional experience, employment status and professional education (MW, PHN, constituted PHN), and user group serviced (pregnant, lactating or non‐pregnant young women).

Questions regarding clinical practice such as dietary advice in general, iodine and other nutrients were inspired by previous research (Arrish et al., 2016; Guess et al., 2017; Lucas et al., 2014) and with reference to the National Guidelines for antenatal care (Norwegian Directorate of Health, 2018). Clinical practice was described via questions using a Likert 5‐point scale ranging from *no extent* to a *very great extent*. The following topics were examined: extent of providing dietary guidance, prioritising of dietary guidance over other guidance, communicating the importance of diet for the foetus/infant, dietary assessment for a specific food, recommendation of specific nutrients and supplements and advice on sufficient intake of iodine sources, barriers to guidance and extent of nutritional training both during and after professional training. Likewise, detailed knowledge of iodine topics was expressed via five questions concerning iodine and the health importance of it, with fixed categories spanning both correct and wrong answers, and ‘do not know’. Topics concerning iodine were national recommended intakes, dietary sources, health importance to foetus/infant, and vulnerable groups according to diet and current national iodine status. Most of the knowledge questions were, with permission, adapted from a pilot version of the questionnaire by Henjum et al. (2018) based on previous studies (Combet et al., 2015; Lucas et al., 2014).

Four MWs and PHNs commended on the questionnaire before distributing the survey, after which a few changes were made. NSD—the Norwegian Centre for Research Data was responsible for the technical performance and collection of the survey.

### 2.3 Statistical analyses

All statistical analyses were conducted using STATA (version 16.0; StataCorp). Demographic data are presented as frequencies and percentages, and mean, min‐max and standard deviation (SD).

Demographic information and clinical practice with related topics were reported for two (MWs and PHNs) or three groups (MWs and PHNs caring for lactating mothers and MWs in antenatal care). T‐test, Mann Withney U Test or Chi‐square Test were used to test for differences between MWs and PHNs and, for some questions, further separated into pregnant women or lactating women. In the analyses, the five categories on the Likert scale were recoded into three categories: *not at all/to a small extent, to some extent* and *a great extent/a very great extent*. Missing numbers were reported in tables.

Further, we investigated the bivariate relationships between the ability to give specific dietary recommendations to ensure adequate iodine nutrient intake and such factors as a profession, demography, dietary clinical practice and education, and iodine knowledge. Correlations between to ordinal variables were examined using Spearman's Rank‐Order Correlation. We applied Mann Withney U Test for comparing the ordinal data of two groups, and the Kruskal Wallis Test for more than two groups.

*p* ≤ 0.05 was considered statistically significant.

### 2.4 Ethical considerations

The Norwegian Regional Committee for Medical and Health Research Ethics (REC North) approved the study (#2017/816). Participants received written information about the study. Information given by participants was evaluated as non‐personally identifiable and thus, no written consent was required. The present study was conducted in accordance with the Helsinki Declaration.

## 3 RESULTS

### 3.1 Participant's characteristics

Table 1 summarises the personal and employment characteristics of the study group. The mean age was ~46 years old for both MWs and PHNs, and the age distribution was similar (*p* = 0.34). Average years of clinical experience were 15 years among the MWs and 10 years for PHNs (*p* < 0.01). A majority of the MWs (59%) had a position in a maternity ward, 24% in antenatal care and 16% worked in a combined position in a maternity unit and antenatal care. Almost all MWs cared for both pregnant and lactating women. Among the PHNs, 13% of the positions were filled by constituted PHNs. Most PHNs worked within the public health centres' diverse range of services spanning from infants to adolescents. Of relevance, 80% of the PHNs provided services to lactating mothers and 75% to young adolescent females (Table 1).

**TABLE 1:** Characteristics of midwives and public health nurses in the study group (*n* = 282).

	Midwives (*n* = 128)		Public health nurses (*n* = 154)		*p*‐value
	Mean (SD) or *n* (%)	Min‐max	Mean (SD) or *n* (%)	Min‐max	
Age					
Years	46.3 (9.6)	26–65	45.8 (8.9)	26–66	0.34^a^
≤30 years	7 (5.5)		6 (3.8)		0.47^b^
31–40 years	30 (23.5)		38 (24.7)		
41–50 years	48 (37.5)		64 (41.3)		
≥50 years	43 (33.5)		46 (29.7)		
Year of education^b^					
Before 1990	14 (10.9)		5 (3.7)		<0.01^b^
1990–1999	33 (25.8)		27 (19.8)		
2000–2009	48 (37.50		50 (36.8)		
2010 or later	33 (25.8)		53 (39.7)		
Work experience					
Years	14.7 (9.2)	0–36	10.3 (7.8)	0–38	0.01^a^
<1–9 years	48 (37.5)		85 (54.8)		<0.01^b^
10–19 years	40 (31.3)		45 (29.1)		
20–29 years	32 (25.0)		23 (14.8)		
30 ≥ years	8 (6.2)		2 (1.3)		
Employment					
Part‐time	41 (34.7)		44 (28.6)		0.02^b^
Full‐time	77 (65.3)		110 (71.4)		
Healthcare institution					
Antenatal care^c^	31 (24.2)				<0.01^b^
Maternity wards^d^	76 (59.4)				
Combined positions^e^	21 (16.4)				
Health Care Clinic children^f^			22 (14.3)		
Other services^g^			36 (23.3)		
Combined positions^h^ ^,^ ^i^			96 (62.4)		
Caring for…					
Adolescents/young women			116 (75.4)		
Pregnant women	123 (96.1)				
Lactating women	118 (92.9)		123 (79.9)		<0.01^b^

^a^ T‐test.

^b^ MW; Mann Withney test between midwives(MWs) and public health nurses (PHNs).

^c^ Constituted public health nurses (PHN) are not included in the numbers for PHN.

^d^ Midwives providing services in antenatal care.

^e^ Midwives providing services at labour/postnatal wards, a midwife‐led unit or outpatient clinic.

^f^ Midwives providing services at both footnotes ‘^c^’ and ‘^d^’.

^g^ Public health nurses providing services at healthcare clinics for children age 0–5.

^h^ Public health nurses providing services at school health services, for youth, students or refugees, or combined.

^i^ Public health nurses providing services at both footnotes ‘^g^’ and ‘^h^’.

### 3.2 Providing dietary guidance

Table 2 shows clinical practice regarding dietary advice to pregnant and lactating women. The majority of MWs and PHNs (83%–89%) stated that they provided dietary guidance to pregnant or lactating women *to a great extent* or *some extent*. The majority of the MWs (~75%) and almost half of the PHNs reported prioritising dietary guidance over other guidance. Furthermore, a majority of both MWs (~70%) and PHNs (~80%) communicated the importance of diet for the foetus or infant *to some* or *to a great extent*. These topics of guidance or information were emphasised more towards pregnant women than towards lactating women. Regarding lactating women, MWs prioritised dietary guidance over other guidance to a greater extent than PHNs (*p* = 0.02), but otherwise, there were no significant differences in practice (Table 2).

**TABLE 2:** Midwives' and public health nurses' clinical practice related to maternal dietary advice.

	Women	Midwives		Public health nurses	MW verus PHN
		Pregnant	Lactating	Lactating	Lactating
	*n*	123	119	123	*p*‐value^b^
	Categorical answers^a^	%	%	%	
In your clinical practice, do you^c^					
Provide dietary guidance	No extent/to a small extent	18.2	15.5	11.1	0.31
	To some extent	22.8	37.9	50.9	
	To a great extent	59.0	46.6	38.0	
	Missing (*n*)	*1*	*3*	*7*	
Prioritise dietary guidance over other guidance	No extent/to a small extent	23.3	32.7	52.5	0.02
	To some extent	44.2	57.5	36.4	
	To a great extent	32.5	9.8	11.0	
	Missing (*n*)	*3*	*6*	*5*	
Communicate the importance of diet for the foetus'/infant's growth and development	No extent/to a small extent	22.5	27.4	19.5	0.58
	To some extent	35.0	47.0	57.7	
	To a great extent	42.5	25.6	22.8	
	Missing (*n*)	*3*	*2*	*5*	
Challenges in counselling related to living habits towards pregnant/lactating women:					
Lack of systematic tools for assessing dietary intake	Not at all/to a small extent	28	22.5	9.2	<0.01
	To some extent	34	27.5	27.5	
	To a great exent	38	50	63.3	
	Not relevant (*n*)	*11*	3	*4*	
	Missing (*n*)	*12*	*18*	*10*	
Lack of time	Not at all/to a small extent	50	51.5	27.0	<0.01
	To some extent	31.3	30.3	34.2	
	To a great exent	16.7	18.2	38.8	
	Not relevant (*n*)	*14*	*5*	*2*	
	Missing (*n*)	*13*	*19*	*10*	

^a^ Five categories originally were merged to three from: not at all, small extent, some extent, great extent.

^b^ MW; Mann Withney U test between midwives (MWs) and public health nurses (PHNs) working with lactating mothers.

^c^ *‘… communicate to lactating women/or to pregnant women as preparation for lactational period about’*.

A majority of MWs (~75%) reported a lack of systematic tools to assess dietary intake among both pregnant and lactating women. Addressing lactating women, the lack of such tools was even more prominent among PHNs (91%) compared to MWs (*p* < 0.01). Also, a lack of time to provide guidance on living habits was challenging for half of the MWs providing both pregnancy and lactation advisory services and was most prominent for the PHNs (73%) who addressed lactating women (*p* < 0.01, Table 2).

### 3.3 Addressing iodine in dietary guidance

**FIGURE 2:**
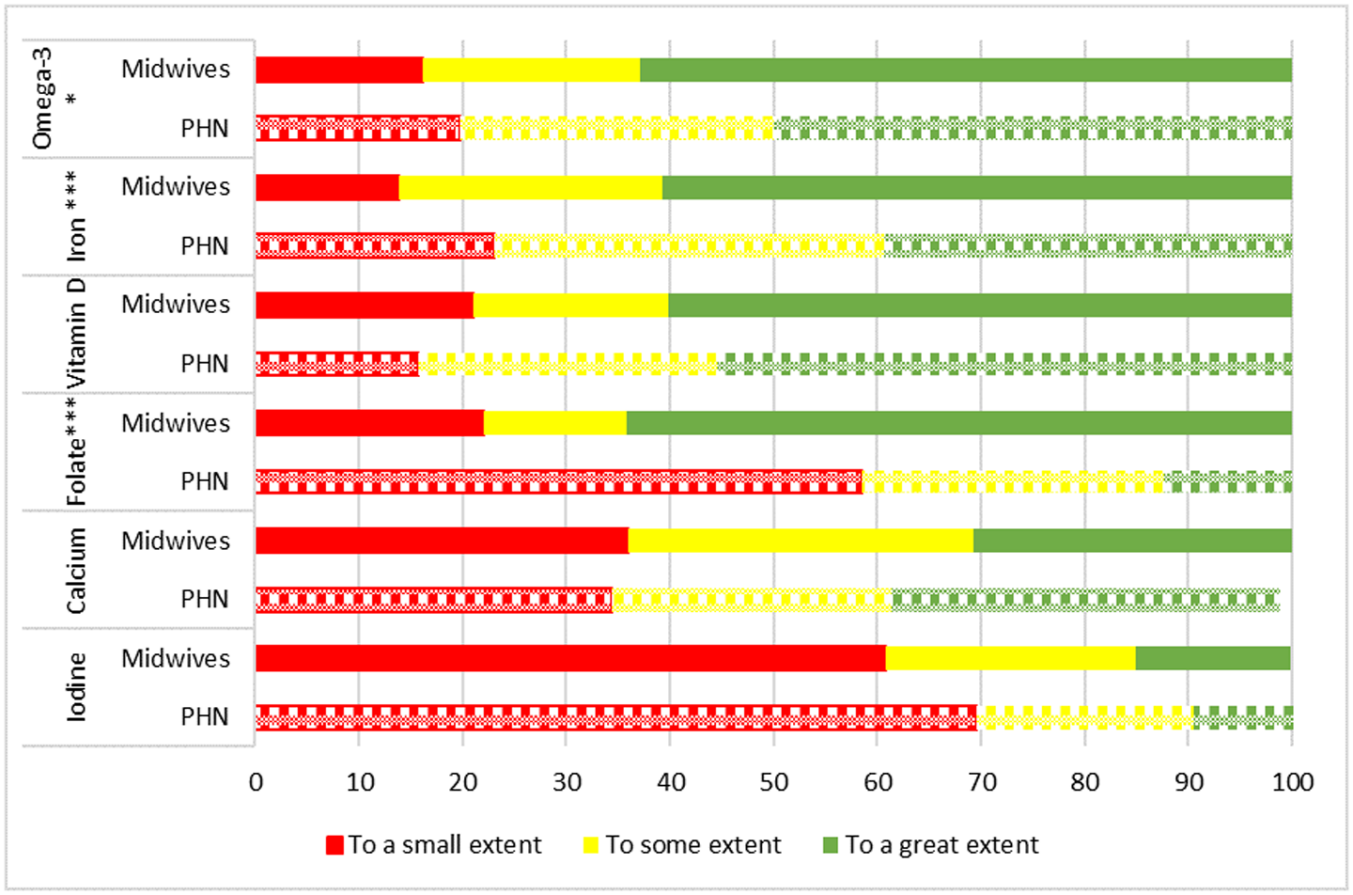
Do you provide specific dietary recommendations to ensure adequate nutrient intake? Public health nurses; PHN. PHN *p*‐value; * < 0.05, ** < 0.01; *** < 0.001.

**FIGURE 3:**
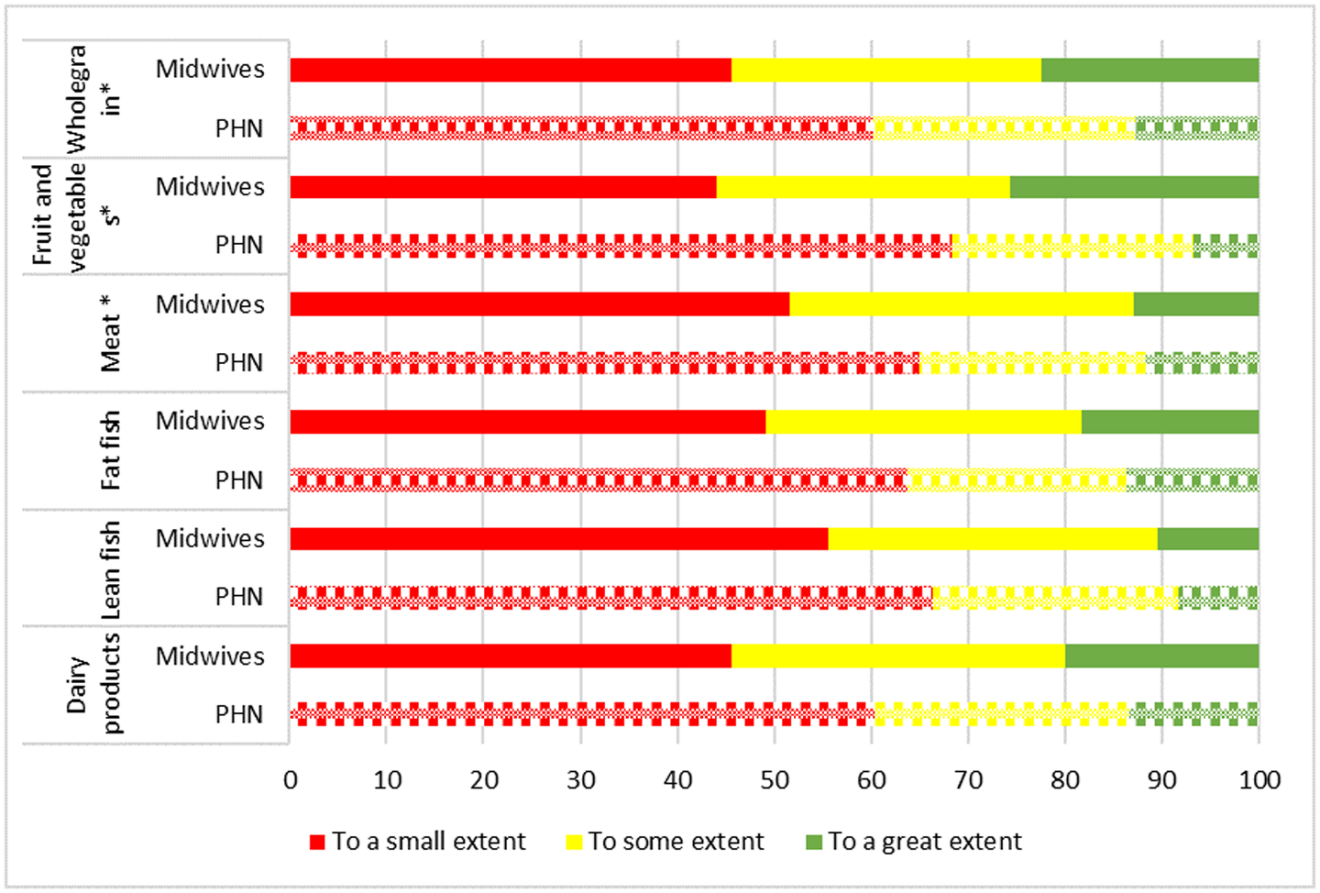
Do you assess how often/how much food the woman eats to get an impression of adequate various nutrient intake? Public health nurses; PHN. PHN *p*‐value; * < 0.05, ** < 0.01; *** < 0.001.

Several questions addressed iodine‐related issues in dietary guidance (Table 3, Figures 2 and 3). When giving dietary guidance to pregnant or lactating women, the majority of both MWs (61%) and PHNs (70%) did *not* recommend *or to a small extent* recommended women specifically to ensure adequate iodine intake (*p* > 0.05, Figure 2). Compared to iodine, both MWs and PHNs put greater emphasis on dietary recommendations to specifically ensure an adequate intake of other nutrients such as calcium, folate, iron and omega‐3 fatty acids and vitamin D (Figure 2). MWs significantly emphasised folate, iron and Omega‐3 more than PHN (*p* < 0.05, Figure 2).

**TABLE 3:** Midwives' and public health nurses' clinical practice related to iodine.

Lifestyle	Categorical answers	Midwives	Public health nurses^a^	*p*‐value	Missing
		(*n* = 128)	(*n* = 123)		
		%	%		
Do you provide information on what type of food to eat, to get enough of each specific nutrient^b^	Not at all/to a small extent	14.3	19	0.05^c^	2
	To some extent	38.1	51.6		
	To a great exent	47.6	29.4		
Provide special advice to those who rarely or never eat fish	Yes	51.6	57,1	0.53^d^	5
	No	48.4	42.9		
Provide special advice to those who rarely or never drink milk	Yes	46.4	63	0.63^d^	6
	No	53.6	37		
Recommend iodine supplementation during pregnancy	Rarely or never	59.8			16
	By indication	34.9			
	Routinely	5.3			
Recommend iodine supplementation during lactating period	Rarely or never	65.2	64.9	0.85^c^	28
	By indication	33	30.7		
	Routinely	1.8	4.4		

^a^ Public health nurses working with lactating women.

^b^ Five categories originally merged to three; not at all, small extent, some extent, great extent and very great extent.

^c^ Mann–Whitney U test.

^d^ Chi‐Square test.

According to the advice about ensuring a nutritious diet, the level of simultaneous advice on what specifically to eat was high (80%, Table 3). Assessing dietary items to gain an impression of whether nutrient intakes were satisfactory was practised more frequently for other items than typical iodine sources such as milk/dairy and lean fish (Figure 3). For these iodine sources, no differences across MWs and PHNs were observed (*p* > 0.05). Furthermore, approximately half of the MWs (~50%) and slightly more PHNs (~60%) provided specific advice to those who rarely or never ate lean fish, or drank milk (Table 3). Additionally, fewer MWs and PHNs recommended iodine supplements by indication. However, approximately 65% of both professions stated that they rarely or never gave recommendations for iodine as a supplement (Table 3).

### 3.4 Iodine knowledge and dietary education among the participants

The descriptive data about iodine knowledge and dietary education is presented in Table 4. Concerning national iodine recommendations for pregnant women, 29% of the MWs and 22% of the PHNs were aware of the recommendation. Overall, 46% recognised iodine deficiency among pregnant and lactating women as a current problem. Around half of the participants correctly identified the most important dietary iodine sources, a lack of fish and seafood (70%) and milk and dairy products (49%) in the diet were identified with iodine deficiency. Around 33% of the participants knew that iodine is important for foetal development, 50% that iodine is of importance for normal growth and development in children and 67% acknowledged the importance for normal metabolism (Table 4).

**TABLE 4:** Midwives' and public health nurses' iodine knowledge and education‐related nutrition.

	Midwives	Public health nurses	*p*‐value	Missing
	(*n* = 128)	(*n* = 123)		
	%	%		
Knowledge questions				
Current national iodine recommendations				
National recommendation/175 μg per day	29.0	22.0	0.47^a^	32
WHO recommendation/250 μg per day	5.3	5.0		
Do not know/remember/wrong answer 100 μg per day	65.7	73.0		
Current iodine status in Norway among pregnant/lactating women				
Too low intake is a current problem	43.1	48.5	0.39^a^	30
Do not know/too high intake is a problem/low intake is not a problem	56.9	51.5		
Most important dietary iodine sources				
Milk and dairy products	48.2	63.5	0.06^a^	34
Fish and seafood	68.8	66.4	0.52^a^	34
Eggs	43.7	40.2	0.50^a^	34
Supplement	42.9	40.2	0.37^a^	34
Vulnerable to iodine deficiency are^b^				
Those with a less satisfactory intake of milk and dairy products				
Yes	44.6	53.2	0.13^a^	0
Not chosen				
Those with a less satisfactory intake of fish and seafood				
Yes	69.4	70.6	0.92^a^	0
Not chosen	30.6	29.4		
Iodine is important for				
Normal foetal development				
Yes	35.9	30.5	0.34^a^	0
Not chosen	64.1	69.5		
Child growth and development				
Yes	48.7	52	0.62^a^	0
Not chosen	51.3	48		
Maintaining a normal metabolism				
Yes	67.9	66.2	0.76^a^	0
Not chosen	32.1	33.8		
Education				
Did/do you receive adequate dietary and nutritional knowledge through your^c^				
Professional training				
No extent/to a small extent	41.4	35	0.21^d^	15
To some extent	45.6	47.9		
To a great extent	13	17.1		
Clinical practice				
No extent/to a small extent	80.3	47.9	<0.01^d^	14
To some extent	18.8	47		
To a great extent	0.9	5.1		

^a^ Chi; chi‐square test.

^b^ Multiple answers possible, only correct answer prompted.

^c^ Five categories originally were merged to three from: not at all, small extent, some extent, great extent and very great extent.

^d^ MW; Mann–Withney U test.

About 40% of the participants *to a small* or *no extent* received adequate knowledge about diet and nutrition through their professional training. The majority of MWs (~80%) and half of the PHNs reported that they *to a small* or *no extent* were offered sufficient professional courses or updated information about the topic diet and nutrition during their current practice. However, the results show that a larger proportion of PHNs received training in this particular topic compared to the MWs (*p* < 0.01, Table 4).

### 3.5 Variables associated with iodine recommendations

Univariate relationships with MWs' and PHNs' degree of providing specific recommendations to ensure adequate iodine nutrient intake and selected variables are shown in Table 5.

**TABLE 5:** Associations between selected factors and the question *‘When you give dietary advice, do you specifically recommend iodine as a nutrient’.*

Group variables	Category	*n*	Mean rank	Rank sum	Spearman rho	*p*‐value
Age group (years)	≤30	9		949.5		0.32^a^
	31‐40	59		6783.5		
	41–50	92		10,514		
	≥51	78		10,194		
Clinical experience (years)	≤5	62		7145.5		0.61^a^
	6‐10	49		5430.5		
	11–20	74		8940		
	21–30	47		6099		
	≥31	6		826		
Work field	MW antenatal care	50	71.6			<0.01^b^
	MW maternity wards	70	52.6			
	MW antenatal care	50	101.9			<0.01^b^
	PHN postnatal care	118	77.1			
	MW maternity wards	70	93.2			0.49^b^
	PHN postnatal care	118	95.3			
Advice about specific food the women must eat to get enough of each nutrient^e^		238			0.38	<0.01^c^
Assess how often/how much food the woman eats to get an impression of adequate various nutrient intake^e^						
Lean fish	Yes	234			0.49	<0.01^c^
Milk and dairy products	Yes	234			0.42	<0.01^c^
Give special advice to those who never drink milk	Yes	131	134.2			<0.01^b^
	No	104	97.6			
Give special advice to those who never eat fish	Yes	133	133.5			<0.01^b^
	No	102	97.9			
Recommend iodine supplementation during pregnancy^e^		132			0.50	<0.01^c^
Recommend iodine supplementation during lactating period^e^		213			0.50	<0.01^c^
Communicate the importance of diet for foetal growth and development during pregnancy		146			0.35	<0.01^c^
Communicate the importance of diet for the infant's growth and development during the lactating period		234			0.28	<0.01^c^
Did you receive adequate dietary and nutritional knowledge through your^d^						
Professional training^f^		227			0.11	0.10^c^
Clinical practice		238			0.04	0.50^c^
Knowledge: most important iodine sources						
Milk and dairy products	Yes	117	13456.5			<0.01^c^
	Not chosen	121	14984.5			
Fish and seafood	Yes	121	14,359			<0.01^c^
	Not chosen	126	16,269			
Knowledge: vulnerable groups for iodine deficiency						
Those who do not eat fish and seafood	Yes	150	19,062			0.02^c^
	Not chosen	88	9379			
Those who do not drink milk or dairy products	Yes	134	14,924			0.03^c^
	Not chosen	104	13,517			
Knowledge: current iodine status in Norway among pregnant/lactating women	Low iodine intake/correct answer	104	124.3			<0.01^c^
	Do not know/remember/high iodine intake	136	92.7			

^a^ KW, Kruskal‐Wallis H Test.

^b^ MW, Mann Withney between midwives (MWs) and public health nurses (PHNs).

^c^ S‐rho, Spearman's Rank‐Order Correlation among all MWs and PHNs as a group together.

^d^ Categorical answers: not at all, to a small extent, to some extent, to a great extent and to a very great extent.

^e^ Categorical answers: rarely or never, by indication, routinely.

^f^ Constituted public health care nurses (not PHN trained) were excluded.

A significant association between pregnant and lactating women and the degree of *dietary recommendations of iodine intake* was seen across services (*p* < 0.01). The ability to give dietary iodine recommendations was significantly higher for MWs working in antenatal care compared to MWs both in maternity wards and PHNs (*p* < 0.01). No significant differences were observed between MWs in maternity wards and the PHNs (*p* > 0.05) (Table 5).

A significant correlation (*r* 0.38, *p* < 0.01) occurred between giving *dietary recommendations for iodine* and providing advice on what type of food a woman must eat to ensure nutrient intake. Corresponding correlations were observed for assessing whether the woman's intake of both lean fish (*r* 0.49, *p* < 0.01) and milk/dairy (*r* 0.42, *p* < 0.01) were satisfactory. Also, giving *dietary recommendations for iodine* was associated with offering specific advice to women who never ate fish (*p* < 0.01) or drank milk (*p* < 0.01) (Table 5). Recommendations for iodine supplements during pregnancy and the lactating period showed a significant positive association with *dietary recommendations for iodine* (*r* 0.50, *p* < 0.01). Communicating the importance of diet for the growth and development of the foetus or infant was correlated with *dietary recommendations of iodine* (*r* 0.35, *p* < 0.01, Table 5).

Knowledge about milk/dairy and fish/seafood as important iodine sources, vulnerable groups of those not consuming these dietary items, and national iodine recommendations and low iodine status among pregnant/lactating women were factors associated with the ability to give *dietary recommendations for iodine* (*p* < 0.05).

There was no association or correlation between *dietary recommendations* concerning *iodine* and age, clinical experience or whether receiving adequate dietary and nutritional education (*p* > 0.05).

### 3.6 Results for PHNs advising young fertile women

A majority of PHNs who served young fertile women stated that, when in contact, they offered dietary guidance *to a great extent* (71%) or *to some extent* (26%). Around half of them provided special advice to those who rarely or never ate fish (52%) or drank milk (50%). The majority of the PHNs *to a small* or *no extent* emphasised the importance of establishing optimal lifestyle habits for future pregnancy (61%) (Table 6).

**TABLE 6:** Clinical practice related to advising adolescents and young fertile women (*n* = 116).

In your practice…	Categorical answers	Public health nurses^a^
		%
Provide dietary guidance^b^	No extent/to a small extent	3.6
	To some extent	25.7
	To a great extent	70.8
Provide special advice to those who rarely or never eat fish	Yes	51.8
	No	48.2
Provide special advice to those who rarely or never drink milk	Yes	50.4
	No	49.6
Communicate the importance of establishing optimal living habits for future pregnancy^b^	No extent/to a small extent	61.4
	To some extent	28.1
	To a great extent	10.5

^a^ Missing number: two participants did not answer any of the questions.

^b^ Five categories originally were merged to three from: not at all, small extent, some extent, great extent and very great extent.

## 4 DISCUSSION

### 4.1 Summary of main findings

This is the first study to document Norwegian clinical practice and knowledge of iodine and its importance to reproductive health. The majority of MW and PHN reported clinical practice with dietary guidance and giving high priority to and communication of the importance of diet. These issues seem to be emphasised more towards pregnant women than towards lactating women. However, the practice and awareness of iodine guidance were low and substantially lower compared to other nutrients. Results also indicate poor iodine knowledge among MWs and PHNs. Nevertheless, the ability to give specific dietary iodine recommendations was adequate for MWs working in antenatal care. The participants that ensured satisfactory iodine dietary recommendations took a more active approach by emphasising dietary assessment, specific food sources and vulnerable groups.

### 4.2 Providing dietary guidance

Our study sample reported a clinical practice where they gave dietary guidance, they prioritised dietary guidance over other guidance and stated that they communicated the importance of diet for the foetus and infant. These results corroborate previous work from Australia in which a majority of healthcare professionals felt they were providing sufficient nutritional support to women during pregnancy (Soltani et al., 2017). In other studies, MWs agreed on the importance of nutritional guidance during pregnancy and highly rated their significant role in providing education and advice on nutrition during pregnancy (Arrish et al., 2016; Lee et al., 2018; Othman et al., 2020). These studies also revealed poor specific practice regarding dietary assessment and advice to ensure specific nutritional intake (Arrish et al., 2016; Lee et al., 2018). Our results were consistent with these, namely: specific practice of obtaining dietary assessment and advice to ensure specific nutritional intake was rather poor for both MWs and PHNs in our study, particularly for iodine. Participants frequently reported giving dietary guidance, but without assessing diet, which suggests a low level of personalisation of the guidance and individual adaptation. This might indicate a superficial clinical practice, which can be viewed in the context of previous studies which conclude that women do not receive adequate nutritional guidance during pregnancy and lactation (Charlton et al., 2012; Soltani et al., 2017). We believe this result is linked to other results from our study: both MWs and PHNs felt that a lack of time was a challenge when it came to counselling about living habits, together with a lack of systematic tools when assessing women's dietary intake. These factors, also suggested by others (Arrish et al., 2016; Othman et al., 2020), may limit the extent of advice and partly explain poor personalisation related to dietary guidance.

### 4.3 Addressing iodine in dietary guidance

In our present study, the majority of the participants answered that they provided information on what type of food to eat, to get enough of each specific nutrient. However, both this and giving specific advice to groups with a low intake of fish and milk were more common than paying attention to ensuring both iodine dietary intake and iodine assessment. This supports our findings of poor iodine focus and might indicate that providing special advice to those with a low intake of fish and milk might well be related to other nutrients such as Omega 3, vitamin D or calcium. Similarly, Guess et al. (2017) demonstrate that less than half of healthcare professionals discussed dietary sources of iodine with the women, and less than 10% reported dietary screening of women for iodine deficiency when planning pregnancy, during pregnancy or in the lactating period (Guess et al., 2017). Another study from Australia also indicates that healthcare professionals had low engagement, particularly concerning the provision of advice on fish consumption and iodine (Charlton et al., 2012).

One unanticipated finding was that around half of the participants in our study answered that a lack of fish, milk and dairy products can cause iodine deficiency, yet the majority do not have a clinical practice concerning iodine. This means that they have knowledge about iodine sources, which makes it somewhat surprising that they do not inform the women about it. A possible interpretation is that the participants are not aware that iodine deficiency is a problem in Norway.

Nevertheless, our results concerning low iodine awareness are supported by previous findings that indicate poor clinical practice and knowledge among healthcare professionals in other countries such as Australia (Arrish et al., 2016; Guess et al., 2017; Lee et al., 2018). Lack of iodine knowledge, as revealed in our study, might negatively influence clinical practice. It is known from earlier research that professional nutritional knowledge influences pregnant women's nutritional knowledge, and as a consequence, this can lead to low adherence to nutrition recommendations and healthy living habits (Bouga et al., 2018; Lee et al., 2018).

Together with dietary changes, the low awareness and the lack of guidance on iodine‐specific recommendations compared to other nutrients might explain the iodine deficiency among women of reproductive age and their children (Norwegian National Council for Nutrition, 2016) that has recently emerged in Norway. In 2016, the Norwegian Nutrition Council highlighted the responsibility of healthcare professionals to ensure a satisfactory iodine status for these vulnerable groups (2016). After our current study, in 2018, iodine recommendations were implemented in antenatal guidelines, which might explain the low awareness among our participants. In contrast to iodine, greater emphasis on folate and vitamin D in our study reflects ‘well‐established’ knowledge and routines among healthcare professionals. A good example is the association between folate deficiency and neural tube defects that was not discovered until the 1990s, as a problem that did not previously receive much attention (Milunsky et al., 1989). This shows us that it takes time to adapt to new knowledge within the nutrition field and reproductive health, yet it is important to reduce the time from establishing firm scientific evidence to its implementation into clinical practice.

### 4.4 Variables associated with dietary iodine recommendations

Interestingly, the ability to give specific dietary iodine recommendations was associated with a practice emphasising dietary priority, dietary assessment, giving special advice about iodine‐rich items, recommending iodine supplements, protecting those with little or no intake of lean fish and milk, and communicating dietary foetal or infant impact. Together with iodine knowledge, this proactive clinical practice might reflect an approach built on evidence‐based practice (NIPH, 2021).

We find it worrying that giving dietary recommendations, particularly specific dietary iodine recommendations, to pregnant women seems to be given higher priority than giving recommendations to lactating women. This finding is consistent with De Waards' (2017) systematic review which points out that recommendations concerning the nutrition status of lactating women and its effect on their infants appears to be scarce. Moreover, uncertainty among the professions, MWs and PHNs, about the responsibility to ensure good nutrition among lactating women seems to be prominent. They are caught between two fields, prenatal and postnatal care, together with a shift of focus from the mother to the infant. Norwegian national postpartum guidelines do not mention maternal dietary recommendations and the impact these can potentially have on women and infants (Norwegian Directorate of Health, 2014). In light of Norway's high rate of exclusive breastfeeding, the lack of focus on maternal dietary issues raises concern for the breastfed infant (Myhre et al., 2020).

### 4.5 Young women

Public health nurses in general give dietary guidance and special advice to young women who never or rarely eat fish or drink milk, but clearly fail to emphasise the importance of establishing optimal living habits for future pregnancy. Based on well‐known knowledge about the significant relationship between lifestyle and future pregnancy, we question whether healthcare professionals' responsibility and awareness of dietary guidance for young women are sufficient or clarified. Despite the optimal focus on the present nutrition situation, our study indicates that PHNs to a minor extent convey the importance of current nutrition status in relation to future outcomes. The responsibility to protect pre‐pregnancy health lies with the general practitioners, together with PHNs. ‘Too little too late’ could be a possible explanation for the iodine deficiency among pregnant women. Increased attention on young women might prevent iodine deficiency when they become pregnant later in life, as suggested by Lee et al. (2018).

### 4.6 Education and knowledge about iodine

The insufficient basic education and lifelong training about dietary and nutrition issues provided through clinical practice and the professional training system in Norway are worrying. Similarly, several studies from other countries show a lack of nutrition training among healthcare professionals, a lack of time and resources, and gaps in knowledge and confidence regarding nutritional health in clinical practice, especially iodine. (Arrish et al., 2014; Bouga et al., 2018; Charlton et al., 2012; Guess et al., 2017; Lucas et al., 2014; Othman et al., 2020) All this might constitute barriers to acknowledging MWs and PHNs role in this important topic.

The fact that iodine deficiency in Norway was not recognised as a problem before 2016 may explain the lack of knowledge in both training systems and clinical practice. After the release of the report by the Norwegian National Council for Nutrition (2016), more attention was expected to be paid to this problem. Authorities play a key role in providing updated information through revised and new guidelines. Iodine deficiency has thus gained more attention since our data collection. Guidelines have been revised and fertile women, as well as infants, have been added as vulnerable groups (Norwegian Directorate of Health, 2018) However, the report from 2016 (Norwegian National Council for Nutrition, 2016) is not included as a reference in either the guidelines for antenatal care or the guidelines for healthcare clinics (0–5 year‐olds) (Norwegian Directorate of Health, 2017, 2018). More worryingly, the postnatal care guidelines, targeting both MWs and PHNs, have not been revised concerning this topic (Norwegian Directorate of Health, 2014). We are concerned that the increased focus on iodine by research and health authorities, targeting healthcare professionals, has not yet been effective enough to change clinical practice.

In addition to the authorities' management of the topic, each clinical health and educational institution has a responsibility to convey and distribute new knowledge. According to the Directorate of Health, all health institutions are obliged to comply with applicable laws and regulations by providing adequate knowledge and skills to every employee within their specific field (Forskrift om ledelse og kvalitetsforbedring i helsetjenesten, 2016). A study by Othman et al. actually concludes that midwives benefited from receiving up‐to‐date training in a pre‐post intervention study (Othman et al., 2020).

Minimal curricula on the topic of nutrition during training could explain the lack of knowledge and confidence towards dietary and nutrition guidance, as previously suggested by others (Arrish et al., 2016). Likewise, maternal nutrition education has not been emphasised in either MWs' or PHNs' study programmes at our institution, according to personal communication at our university, which is the primary education institution for the target healthcare personnel in northern Norway. Reasons for absence from the agendas of education institutions could be less focus on nutritional education in general, or that it is expected that MWs' and PHNs' work is evidence‐based. Possessing this competence includes obtaining new knowledge, guidelines or research by themselves (NOU, 2018:2). This is also in accordance with the Norwegian ‘Health Personnel Act’ whereby every individual working within healthcare has an independent professional responsibility to maintain disciplinary practice (Helsesersonelloven, 1999).

### 4.7 Strengths and limitations

This study has several strengths; it is the first study of healthcare personnel's clinical practice concerning iodine in a Norwegian context. All public clinics in northern Norway were invited to participate. However, due to the indirect recruitment method, with an internal distribution of pre‐information, invitation and reminders relying on the clinic's benevolence, the number of invitations that were received is uncertain. At best, the response rate might be higher than reported. Nonetheless, our low participation rate may have introduced non‐response bias and limited generalisation, especially for MWs in maternity wards and PHNs. A lack of response might reflect their perception that the survey topics are of less relevance, interest, and priority. Our participation rate is nonetheless in line with declining trends in studies elsewhere (Wu et al., 2022). We used a questionnaire adapted from a pilot version by Henjum et al. (2018), and unfortunately, knowledge about non‐animal sources of iodine was, therefore, not listed. Another weakness is the lack of multivariable analyses and the lack of a validated questionnaire addressing clinical practice, yet strengthened by derivations from national guidelines, previous research or reused from other studies. Also, an information bias linked to a self‐reported survey cannot be excluded as it presents oneself in a better light and with a better understanding of questions. This survey was conducted in 2017–2018, yet a follow‐up is relevant and can serve as a baseline study for evaluating the effect of campaign awareness and the aftermath of implementing iodine guidelines, and whether the increased focus at a national level has been effective. Despite these weaknesses, the results indicate weak practice regarding the iodine issue in relation to reproductive health.

## 5 CONCLUSION

In their clinical practice, MWs and PHNs are in a unique position to enhance public awareness of iodine, dietary sources and how to safeguard daily intake. However, the results of our study indicate that MWs and PHNs had a lack of clinical practice and knowledge of iodine concerning fertile women and infants, especially among lactating women. Increased focus on iodine and specific dietary guidelines during MWs' and PHNs' professional training and through clinical practice is necessary.

## 6 RELEVANCE TO CLINICAL PRACTICE

In light of the current iodine deficiency and lack of iodine knowledge among reproductive target groups, especially among lactating mothers, it is important that healthcare providers such as midwives and public health nurses enhance their clinical practice and knowledge in relation to iodine intake and dietary sources, to convey and prevent possible negative outcomes for women, foetus and infant.

## AUTHOR CONTRIBUTIONS

PhD student Maren Johnsen performed the analysis, drafted the manuscript, in close collaboration with Associate professor (PhD) Solrunn Hansen. Professor Tonje Braaten was the statistician on the author team and verified the analyses and results. Professor Guri Skeie and associate professor (PhD) Hilde Laholt contributed and commented on the writing of the manuscript. Associate professor (PhD) Hansen initiated the project.

## FUNDING INFORMATION

The Northern Norway Regional Health Authority has financed parts of the research.

## CONFLICT OF INTEREST STATEMENT

No conflict of interest.

## PATIENT OR PUBLIC CONTRIBUTION

Four MWs' and PHNs' commended the questionnaire during the design of the survey, otherwise no patient or public contribution.

## Data Availability

The data that support the findings of this study are available on request from the corresponding author, Solrunn Hansen.

## References

[nop21675-bib-0001] Aakre, I. , Morseth, M. S. , Dahl, L. , Henjum, S. , Kjellevold, M. , Moe, V. , Smith, L. , & Markhus, M. W. (2020). Iodine status during pregnancy and at 6 weeks, 6, 12 and 18 months post‐partum. Maternal and Child Nutrition, 17, e13050. 10.1111/mcn.13050 32602197 PMC7729798

[nop21675-bib-0002] Andersson, M. , de Benoist, B. , Delange, F. , & Zupan, J. (2007). Prevention and control of iodine deficiency in pregnant and lactating women and in children less than 2‐years‐old: Conclusions and recommendations of the Technical Consultation. Public Health Nutrition, 10(12A), 1606–1611. 10.1017/S1368980007361004 18053287

[nop21675-bib-0003] Andersson, M. , Karumbunathan, V. , & Zimmermann, M. (2012). Global iodine status in 2011 and trends over the past decade. Journal of Nutrition, 2012(142), 744–750. 10.3945/jn.111.149393 22378324

[nop21675-bib-0004] Arrish, J. , Yeatman, H. , & Williamson, M. (2014). Midwives and nutrition education during pregnancy: A literature review. Women and Birth, 27(1), 2–8. 10.1016/j.wombi.2013.02.003 23562582

[nop21675-bib-0005] Arrish, J. , Yeatman, H. , & Williamson, M. (2016). Australian midwives and provision of nutrition education during pregnancy: A cross sectional survey of nutrition knowledge, attitudes, and confidence. Women and Birth, 29(5), 455–464. 10.1016/j.wombi.2016.03.001 27020228

[nop21675-bib-0006] Berg, V. , Nøst, T. , Skeie, G. , Thomassen, Y. , Berlinger, B. , Veyhe, A. S. , Jorde, R. , Odland, J. Ø. , & Hansen, S. (2017). Thyroid homeostasis in mother–child pairs in relation to maternal iodine status: The MISA study. European Journal of Clinical Nutrition, 71, 1002–1007. 10.1038/ejcn.2017.83 28537582 PMC5543254

[nop21675-bib-0007] Bouga, M. , Lean, M. E. , & Combet, E. (2018). Iodine and pregnancy—A qualitative study focusing on dietary guidance and information. Nutrients, 10(4), 408. 10.3390/nu10040408 29587423 PMC5946193

[nop21675-bib-0008] Brantsæter, A. , Abel, M. , Haugen, M. , & Meltzer, H. (2013). Risk of suboptimal iodine intake in pregnant Norwegian women. Nutrients, 5(2), 424–440. 10.3390/nu5020424 23389302 PMC3635203

[nop21675-bib-0009] Bryant, J. , Waller, A. E. , Cameron, E. C. , Sanson‐Fisher, R. W. , & Hure, A. J. (2019). Receipt of information about diet by pregnant women: A cross‐sectional study. Women and Birth, 32(6), e501–e507. 10.1016/j.wombi.2018.12.005 30559008

[nop21675-bib-0010] Candido, A. C. , Morais, N. S. , Dutra, L. V. , Pinto, C. A. , Franceschini, S. , & Alfenas, R. C. G. (2019). Insufficient iodine intake in pregnant women in different regions of the world: A systematic review. Archives of Endocrinology and Metabolism, 63(3), 306–311. 10.20945/2359-3997000000151 31340241 PMC10522210

[nop21675-bib-0011] Charlton, K. , Yeatman, H. , Lucas, C. , Axford, S. , Gemming, L. , Houweling, F. , Goodfellow, A. , & Ma, G. (2012). Poor knowledge and practices related to iodine nutrition during pregnancy and lactation in Australian women: Pre‐ and post‐iodine fortification. Nutrients, 4(9), 1317–1327. 10.3390/nu4091317 23112919 PMC3475241

[nop21675-bib-0012] Combet, E. , Bouga, M. , Pan, B. , Lean, M. E. J. , & Christopher, C. O. (2015). Iodine and pregnancy—A UK cross‐sectional survey of dietary intake, knowledge and awareness. The British Journal of Nutrition, 114(1), 108–117. 10.1017/S0007114515001464 26005740

[nop21675-bib-0013] Forskrift om ledelse og kvalitetsforberding i helse‐ og omsorgstjenesten . (2016). Forskrift om ledelse og kvalitetsforbedring i helsetjenesten. [Regulation on management and quality improvement in health care service]. (FOR‐2016‐10‐28‐1250). https://lovdata.no/dokument/LTI/forskrift/2016‐10‐28‐1250

[nop21675-bib-0014] Garnweidner‐Holme, L. , Aakre, I. , Lilleengen, A. , Brantsæter, A. , & Henjum, S. (2017). Knowledge about iodine in pregnant and lactating women in the Oslo area, Norway. Nutrients, 9(5), 493. 10.3390/nu9050493 28505075 PMC5452223

[nop21675-bib-0015] Groufh‐Jacobsen, S. , Mosand, L. M. , Bakken, K. S. , Solvik, B. S. , Oma, I. , Gjengedal, E. L. F. , Brantsaeter, A. L. , Strand, T. A. , & Henjum, S. (2020). Mild to moderate iodine deficiency and inadequate iodine intake in lactating women in the inland area of Norway. Nutrients, 12(3), 630. 10.3390/nu12030630 32120975 PMC7146631

[nop21675-bib-0016] Guess, K. , Malek, L. , Anderson, A. , Makrides, M. , & Zhou, S. J. (2017). Knowledge and practices regarding iodine supplementation: A national survey of healthcare providers. Women and Birth, 30(1), e56–e60. 10.1016/j.wombi.2016.08.007 27599944

[nop21675-bib-0017] Helse‐ og omsorgstjenesteloven . (2011). Lov om kommunale helse og omsorgstjenester m.m. [Regulation conserning Health and Care in the Municipal etc.,]. (LOV‐2011‐06‐24‐30). Lovdata. https://lovdata.no/dokument/NL/lov/2011‐06‐24‐30#KAPITTEL_3

[nop21675-bib-0018] Helsepersonelloven . (1999). Lov om helsepersonell. [The Health Personnel Act] (LOV‐1999‐07‐02‐64) Lovdata. https://lovdata.no/dokument/NL/lov/1999‐07‐02‐64

[nop21675-bib-0019] Henjum, S. , Abel, M. H. , Meltzer, H. M. , Dahl, L. , Alexander, J. , Torheim, L. E. , & Brantsæter, A. L. (2019). Er inntaket av jod i befolkningen tilstrekkelig? [Is iodine intake adequate in Norway?]. Tidsskrift for Den Norske Legeforening, 139(2), 159–164. 10.4045/tidsskr.18.0319 30698392

[nop21675-bib-0020] Henjum, S. , Brantsaeter, A. L. , Kurniasari, A. , Dahl, L. , Aadland, E. K. , Gjengedal, E. L. F. , Birkeland, S. , & Aakre, I. (2018). Suboptimal iodine status and low iodine knowledge in young Norwegian women. Nutrients, 10(7), 941. 10.3390/nu10070941 30037088 PMC6073112

[nop21675-bib-0021] Henjum, S. , Lilleengen, A. , Aakre, I. , Dudareva, A. , Gjengedal, E. , Meltzer, H. , & Brantsæter, A. (2017). Suboptimal iodine concentration in breastmilk and inadequate iodine intake among lactating women in Norway. Nutrients, 9(7), 643. 10.3390/nu9070643 28640217 PMC5537763

[nop21675-bib-0022] ICM . (2008). ICM international code of ethics for midwives. JMWH, 49(3), 264–265 https://www.internationalmidwives.org/assets/files/definitions‐files/2018/06/eng‐international‐code‐of‐ethics‐for‐midwives.pdf

[nop21675-bib-0023] ICN . (2011). ICN international code of ethics for nursing. In Yrkesetiske retningslinjer for sykepleiere [Rev. ed]. Norsk sykepleierforbund. https://www.nsf.no/etikk‐0/yrkesetiske‐retningslinjer

[nop21675-bib-0024] Jiang, H. , Powers, H. J. , & Rossetto, G. S. (2019). A systematic review of iodine deficiency among women in the UK. Public Health Nutrition, 22(6), 1138–1147. 10.1017/s1368980018003506 30596360 PMC10260541

[nop21675-bib-0025] Lee, A. , Newton, M. , Radcliffe, J. , & Belski, R. (2018). Pregnancy nutrition knowledge and experiences of pregnant women and antenatal care clinicians: A mixed methods approach. Women and Birth, 31(4), 269–277. 10.1016/j.wombi.2017.10.010 29126796

[nop21675-bib-0026] Lucas, C. , Charlton, K. E. , & Yeatman, H. (2014). Nutrition advice during pregnancy: Do women receive it and can health professionals provide it? Maternal and Child Health Journal, 18(10), 2465–2478. 10.1007/s10995-014-1485-0 24748213

[nop21675-bib-0027] Malta, M. B. , Carvalhaes, M. A. , Takito, M. Y. , Tonete, V. L. , Barros, A. J. , Parada, C. M. , & Benício, M. H. (2016). Educational intervention regarding diet and physical activity for pregnant women: Changes in knowledge and practices among health professionals. BMC Pregnancy and Childbirth, 16(1), 1–9. 10.1186/s12884-016-0957-1 27439974 PMC4955265

[nop21675-bib-0028] Milunsky, A. , Jick, H. , Jick, S. S. , Bruell, C. L. , MacLaughlin, D. S. , Rothman, K. J. , & Willett, W. (1989). Multivitamin/folic acid supplementation in early pregnancy reduces the prevalence of neural tube defects. Jama, 262(20), 2847–2852. 10.1001/jama.262.20.2847 2478730

[nop21675-bib-0029] Myhre, J. B. , Andersen, L. F. , & Kristiansen, A. L. (2020). Spedkost 3. Landsomfattende undersøkelse av kostholdet blant spedbarn i Norge, 6 måneder [Spedkost 3. Nationwide dietary survey among infants in Norway, age 6 months]. Folkehelseinstituttet og universitetet i Oslo https://www.fhi.no/publ/2020/spedkost‐3/

[nop21675-bib-0030] Næss, S. , Markhus, M. W. , Strand, T. A. , Kjellevold, M. , Dahl, L. , Stokland, A. M. , Nedrebø, B. G. , & Aakre, I. (2021). Iodine nutrition and iodine supplement initiation in association with thyroid function in mildly‐to‐moderately iodine‐deficient pregnant and postpartum women. The Journal of Nutrition, 151(10), 3187–3196. 10.1093/jn/nxab224 34255063 PMC8485914

[nop21675-bib-0031] NIPH [Norwegian Institute of Public Health]. Folkehelseinstituttet . (2021). Kunnskapsbasert praksis. Folkehelseinstituttet https://www.helsebiblioteket.no/kunnskapsbasert‐praksis

[nop21675-bib-0032] Norwegian Directorate of Health . (2011). Helsedirektoratets kostråd. [Directorate of Health Dietary Guidelines]. Helsedirektoratet (updated 2015). https://www.helsenorge.no/kosthold‐og‐ernaring/kostrad/helsedirektoratets‐kostrad/

[nop21675-bib-0033] Norwegian Directorate of Health . (2014). Nasjonal faglig retningslinje for barselomsorgen – Nytt liv og trygg barseltid for familien. [National Guidelines for postnatal care]. Helsedirektoratet https://www.helsedirektoratet.no/retningslinjer/barselomsorgen

[nop21675-bib-0034] Norwegian Directorate of Health . (2016). Nasjonal faglig retningslinje‐Spedbarnsernæring. [National guideline for infant nutrition]. Helsedirektoratet https://www.helsedirektoratet.no/retningslinjer/spedbarnsernaering

[nop21675-bib-0035] Norwegian Directorate of Health . (2017). Nasjonal faglig retningslinje for det helsefremmende og forebyggende arbeidet i helsestasjon, skolehelsetjeneste og helsestasjon for ungdom. [National guideline for health promotion and preventive work in the child and youth health centres and school health service, 0–20 years]. Helsedirektoratet (updated 2022). https://www.helsedirektoratet.no/retningslinjer/helsestasjons‐og‐skolehelsetjenesten

[nop21675-bib-0036] Norwegian Directorate of Health . (2018). Nasjonal faglig retningslinje for svangerskapsomsorgen. [National Guideline for Antenatal Care]. Helsedirektoratet (updated 2022). https://www.helsedirektoratet.no/retningslinjer/svangerskapsomsorgen

[nop21675-bib-0037] Norwegian National Council for Nutrition. [Nasjonalt råd for ernæring] . (2016). Risiko for jodmangel i Norge‐identifisering av et akutt behov for tiltak. [Risk of iodine deficiancy in Norway‐identifying an urget need for action]. Helsedirketoratet [Norwegian Directorate of Health] http://www.ernaeringsradet.no/wp‐content/uploads/2016/06/IS‐0591_RisikoForJodmangeliNorge.pdf

[nop21675-bib-0038] NOU 2018: 2 . (2018). Fremtidige kompetansebehov I: Kunnskapsgrunnlaget. [Official Norwegian Report NOU 2018:2 Competence in future]. Kunnskapsdepartementet https://www.regjeringen.no/no/dokumenter/nou‐2018‐2/id2588070/

[nop21675-bib-0039] Nyström, H. F. , Brantsæter, A. L. , Erlund, I. , Gunnarsdottir, I. , Hulthén, L. , Laurberg, P. , Mattisson, I. , Rasmussen, L. B. , Virtanen, S. , & Meltzer, H. M. (2016). Iodine status in the Nordic countries—Past and present. Food & Nutrition Research, 60(1), 31969–31915. 10.3402/fnr.v60.31969 27283870 PMC4901513

[nop21675-bib-0040] Othman, S. M. E. , Steen, M. , Fleet, J. A. , & Jayasekara, R. (2020). Healthy eating in pregnancy, education for midwives: A pre‐post intervention study. European Journal of Midwifery, 4, 20. 10.18332/ejm/120004 33537622 PMC7839119

[nop21675-bib-0041] Patriota, E. S. O. , Lima, I. C. C. , Nilson, E. A. F. , Franceschini, S. C. C. , Gonçalves, V. S. S. , & Pizato, N. (2022). Prevalence of insufficient iodine intake in pregnancy worldwide: A systematic review and meta‐analysis. European Journal of Clinical Nutrition, 76(5), 703–715. 10.1038/s41430-021-01006-0 34545212

[nop21675-bib-0042] Soltani, H. , Duxbury, A. , Rundle, R. , & Marvin‐Dowle, K. (2017). Dietary habits and supplementation practices of young women during pregnancy: An online cross‐sectional survey of young mothers and health care professionals. BMC Nutrition, 3(1), 1–15. 10.1186/s40795-017-0137-3 PMC7050751 32153801

[nop21675-bib-0044] World Health Organization . (2007). Assessment of iodine deficiency disorders and monitoring their elimination: A guide for programme managers (3rd ed.). https://apps.who.int/iris/handle/10665/43781

[nop21675-bib-0045] Wu, M.‐J. , Zhao, K. , & Fils‐Aime, F. (2022). Response rates of online surveys in published research: A meta‐analysis. Computers in Human Behavior Reports, 7, 100206. 10.1016/j.chbr.2022.100206

[nop21675-bib-0046] Zimmermann, M. B. (2009). Iodine deficiency in pregnancy and the effects of maternal iodine supplementation on the offspring: A review. AJCN, 89(2), 668 S–672 S. 10.3945/ajcn.2008.26811C 19088150

[nop21675-bib-0047] Zimmermann, M. B. (2011). The role of iodine in human growth and development. Seminars in Cell & Developmental Biology, 22(6), 645–652. 10.1016/j.semcdb.2011.07.009 21802524

[nop21675-bib-0048] Zimmermann, M. B. , Gizak, M. , Abbott, K. , Andersson, M. , & Lazarus, J. H. (2015). Iodine deficiency in pregnant women in Europe. The Lancet Diabetes and Endocrinology, 3(9), 672–674. 10.1016/s2213-8587(15)00263-6 26268907

